# Comparative Analysis of Long-Term Variation Characteristics of SO_2_, NO_2_, and O_3_ in the Ecological and Economic Zones of the Western Sichuan Plateau, Southwest China

**DOI:** 10.3390/ijerph16183265

**Published:** 2019-09-05

**Authors:** Pengguo Zhao, Jia Liu, Yu Luo, Xiuting Wang, Bolan Li, Hui Xiao, Yunjun Zhou

**Affiliations:** 1 Plateau Atmosphere and Environment Key Laboratory of Sichuan Province, College of Atmospheric Science, Chengdu University of Information Technology, Chengdu 610225, China; 2 Heavy Rain and Drought-Flood Disasters in Plateau and Basin Key Laboratory of Sichuan Province, Chengdu 610072, China; 3 Climate Center of Sichuan Province, Chengdu 610072, China; 4 Sichuan Ecological Environment Monitoring Center, Chengdu 610041, China; 5 Guangzhou Institute of Tropical and Marine Meteorology, China Meteorological Administration, Guangzhou 510080, China

**Keywords:** sulfur dioxide, nitrogen dioxide, ozone, Western Sichuan Plateau, long-term variation

## Abstract

Sulfur dioxide (SO_2_), nitrogen dioxide (NO_2_), and ozone (O_3_) are important atmospheric pollutants that affect air quality. The long-term variations of SO_2_ and NO_2_ in 2008–2018 and O_3_ in 2015–2018 in the relatively less populated ecological and economic zones of Western Sichuan Plateau, Southwest China were analyzed. In 2008–2018, the variations in SO_2_ and NO_2_ in the ecological zone were not significant, but Ganzi showed a slight upward trend. SO_2_ decreased significantly in the economic zone, especially in Panzhihua, where NO_2_ changes were not obvious. From 2015 to 2018, the concentration of O_3_ in the ecological zone increased significantly, while the economic zone showed a downward trend. The rising trend of the concentration ratio of SO_2_ to NO_2_ in the ecological zone and the declining trend in the economic zone indicate that the energy consumption structure of these two zones is quite different. The lower correlation coefficients between NO_2_ and O_3_ in the Western Sichuan Plateau imply that the variations of O_3_ are mainly affected by the regional background. The effects of meteorological factors on SO_2_, NO_2_, and O_3_ were more obvious in the economic zone where there are high anthropometric emissions.

## 1. Introduction

In recent decades, China’s economy has developed rapidly. Especially in the 21st century, the emission of artificial air pollutants has increased significantly, and hazy weather is frequent; therefore, the air pollution problem has attracted people’s attention. The Sichuan Basin is one of the areas in China suffering from serious air pollution due to its special weather and topography [1,2,3,4]. Sulfur dioxide (SO_2_), nitrogen dioxide (NO_2_), and ozone (O_3_) are important pollutants that not only affect the generation of atmospheric fine particles and the photochemical and atmospheric acidification processes but also threaten human health [5,6,7,8]. Many previous studies [9,10,11] have carried out quantitative analysis of SO_2_, NO_2_, and O_3_ pollutants, discussed the diurnal and seasonal variation characteristics, and pointed out that SO_2_ and NO_2_ have the lowest concentration in summer due to the wet cleaning effect of precipitation, while O_3_ has the highest concentration as a result of the most active photochemical reactions occurring in spring and summer, indicating that NO_x_ is an important precursor of O_3_.

Several studies have focused on the variations of SO_2_, NO_2_, and O_3_ in the most polluted areas in China, such as the Beijing-Tianjin-Hebei region, North China, and the Yangtze River and Pearl River Deltas. The results show that the concentration of SO_2_ declined sharply in the past decade, especially after 2006, because of the SO_2_ emission reduction requirement in China’s Nineth Five-Year Plan (1996–2000). The concentrations of NO_2_ in large and medium-sized cities of China showed a fluctuating upward trend due to the lack of effective denitration technology [12,13,14,15,16,17]. Due to the late observation time and complicated formation mechanism, few studies have been conducted on the long-term variation characteristics of O_3_. In the past five years, serious O_3_ pollution has appeared in the large cities of China, and O_3_ has replaced PM_2.5_ as the primary pollutant in some cities [18,19,20]. Zhao et al. [21] analyzed the characteristics of six criteria air pollutants in the Sichuan Basin of Southwest China from 2015 to 2017 and pointed out that all air pollutants except O_3_ showed U-shaped annual changes; motor vehicle emissions was the main contributor to O_3_ in the basin, while industrial emission was the main contributor to O_3_ in the Western Sichuan Plateau area. Air quality in many large and medium-sized cities has improved significantly as a result of various environmental policies implemented by the Chinese government [22,23]. The changes of air pollutants in the sparsely populated western plateau in the past decade remain in question.

The Sichuan Provincial Government [24] has designated Aba and Ganzi as ecological zones, and Liangshan and Panzhihua as economic zones based on the industrial structure and gross domestic product (GDP), respectively. They are located in a high-altitude area in the transition from the Sichuan Basin to the Tibet Plateau, called the Western Sichuan Plateau. Due to the sparsely populated land and low emissions of man-made pollutants, this is a clean air background region in southwest China and even the whole country. The study of the long-term variation characteristics of atmospheric pollutants in this region is of great scientific significance for a comparative analysis of pollutants in other regions of China. Based on the daily mass concentration of SO_2_ and NO_2_ from 2008 to 2018 and O_3_ from 2015 to 2018, long-term variations and the basic characteristics of three gaseous air pollutants in the ecological and economic zones of Western Sichuan Plateau were compared and analyzed in this paper.

## 2. Materials and Methods

**Figure 1:**
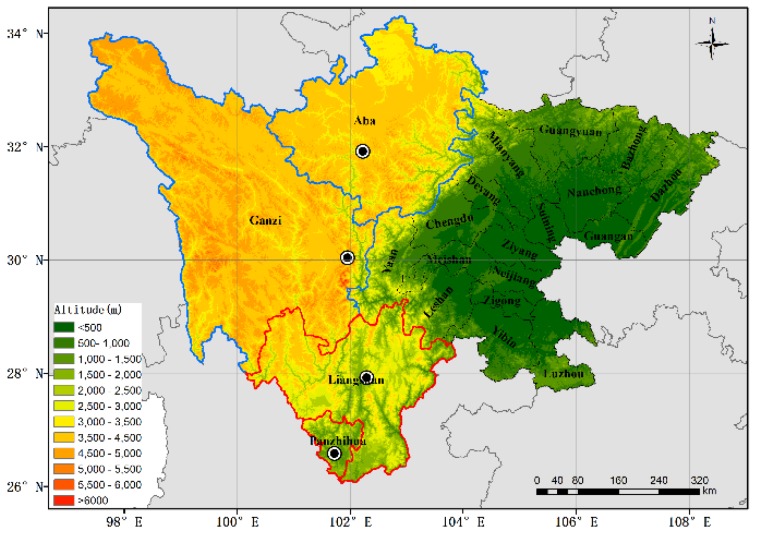
Geographical position of ecological zone (**blue outline**) and economic zone (**red outline**) of Western Sichuan Plateau, the locations of cities are marked by black dots.

The ecological and economic zones of Western Sichuan Plateau are located east of the Tibetan Plateau. The ecological zone includes Aba and Ganzi, and the economic zone includes Liangshan and Panzhihua. The geographical locations of these zones are shown in Figure 1, the area outlined in blue is the ecological zone and the area outlined in red is the economic zone, and the locations of cities are marked by black dots. The average altitude of Aba, Ganzi, Liangshan, and Panzhihua is 3000 m, 4100 m, 1500 m, and 1500 m, respectively.

The air pollutants data used in this paper were collected from Sichuan Ecological Environment Monitoring Center (http://www.scnewair.cn:6112/publish/index.html) [25]. The citywide daily average concentrations of SO_2_ and NO_2_ from 1 January 2008 to 31 December 2018, and the citywide daily maximum eight-hour average concentration of O_3_ from 1 January 2015 to 31 December 2018 were used to analyze the spatiotemporal distribution characteristics of three air pollutants in Western Sichuan Plateau. The mass concentrations of SO_2_, NO_2_, and O_3_ were measured by the ultraviolet fluorescence method (TEI, Model 43i, Thermo Fisher Scientific Inc., Waltham, MA, USA), the chemiluminescence method (TEI, Model 42i, Thermo Fisher Scientific Inc., USA), and the UV spectrophotometry method (TEI, Model 49i, Thermo Fisher Scientific Inc., USA), respectively [21]. The measurements were conducted by multiple national air quality monitoring sites in cities [26]. The daily average concentrations of the three air pollutants released after quality assurance and control by the Sichuan Ecological Environment Monitoring Center based on Technical Guideline on Environmental Monitoring Quality Management HJ 630-2011 (http://kjs.mep.gov.cn) were the same as the daily air quality reports issued by the government. In addition, at least 324 and 25 daily concentrations were required to calculate the average annual concentrations of SO_2_, NO_2_, and O_3_.

The routine meteorological parameters were collected from the National Meteorological Information Center of China [27]. The surface stations are shown by black dots in Figure 1. The monthly mean meteorological parameters included mean monthly average temperature, maximum and minimum monthly average temperature, mean relative humidity, monthly total precipitation, monthly sunshine duration, mean wind speed, and maximum wind speed from January 2008 to December 2017. The data quality is controlled by the National Meteorological Information Center of China and the accuracy rate is close to 100%.

The related statistical data of the basic situation of each city were collected from the statistical yearbook provided by the Sichuan provincial bureau (http://tjj.sc.gov.cn/tjnj). Table 1 shows the region category, area, resident population, urbanization rate, GDP, and possession of civil motor vehicles of the study area at the end of 2016. The urbanization rates of Aba, Ganzi, and Liangshan are relatively low, at 37.86%, 29.26%, and 33.04%, respectively. GDP and the number of vehicles in Liangshan and Panzhihua are obviously higher than in Aba and Ganzi, which means there are more man-made air pollutants in the economic zone (Liangshan and Panzhihua).

## 3. Results and Discussion

### 3.1. Temporal Variations of SO_2_, NO_2_, and O_3_

**Figure 2:**
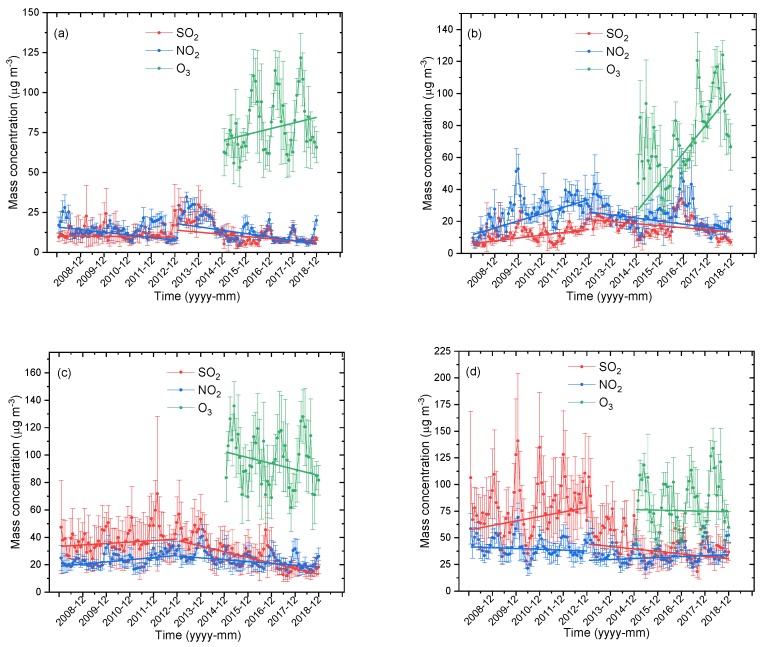
Variations of monthly mean mass concentrations of SO_2_, NO_2_, and O_3_ in (**a**) Aba, (**b**) Ganzi, (**c**) Liangshan, and (**d**) Panzhihua from 2008 to 2018.

The variations of SO_2_ and NO_2_ from 2008 to 2018 and O_3_ from 2015 to 2018 in the ecological and economic zones of Western Sichuan Plateau based on monthly average mass concentration are shown in Figure 2. The variations of SO_2_ and NO_2_ were divided into two periods before and after 2012. SO_2_ and NO_2_ in Aba showed a downward trend before and after 2012, and the downward trend was more prominent after 2012. However, SO_2_ and NO_2_ in Ganzi increased before 2012 and decreased after 2012. O_3_ in the ecological zone of the Western Sichuan Plateau, Aba and Ganzi, showed a significant upward trend, with a rising rate of 0.303 μg·m^−3^/month and 0.154 μg·m^−3^/month, respectively. Ma et al. [28] reported an increasing trend of surface O_3_ in a rural site north of eastern China and indicated that meteorological factors had little influence on long-term O_3_ change, which was completely related to emissions. SO_2_ and NO_2_ in Liangshan showed a trend of first rising and then falling before and after 2012, and the decline of SO_2_ was particularly significant after 2012. SO_2_ in Panzhihua increased significantly before 2012 and decreased after 2012, while NO_2_ showed a slight decline and increase trend before and after 2012. The declining trend of O_3_ in Liangshan was very prominent, with a declining rate of 0.36 μg·m^−3^/month, while the change range of O_3_ in Panzhihua, with the highest urbanization rate, was not obvious. In general, the changes of SO_2_ and NO_2_ in the ecological zone of Western Sichuan Plateau were weak, while the decrease of SO_2_ in the economic zone was very significant and the variation trend of O_3_ in the ecological and economic zones was opposite.

**Figure 3:**
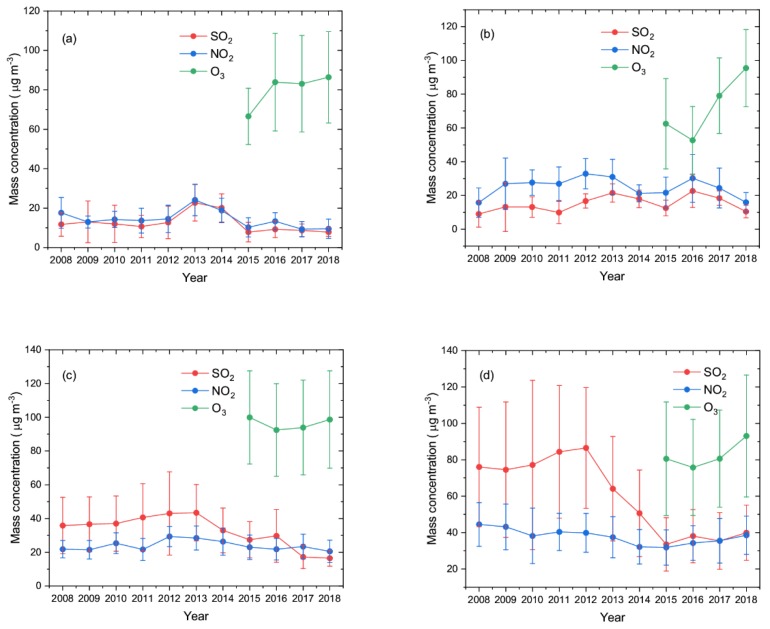
Variations of annual mean mass concentrations of SO_2_, NO_2_, and O_3_ in (**a**) Aba, (**b**) Ganzi, (**c**) Liangshan, and (**d**) Panzhihua from 2008 to 2018.

For further analysis, Figure 3 shows the annual variation trend of SO_2_, NO_2_, and O_3_ in the ecological and economic zones of Western Sichuan Plateau, based on the annual average mass concentrations of SO_2_ and NO_2_ from 2008 to 2018, and O_3_ from 2015 to 2018. The concentrations of SO_2_ and NO_2_ in the ecological area were relatively lower due to the small influence of human activity. The annual variation characteristics of SO_2_ and NO_2_ in Aba were very similar; their annual concentrations remained stable at about 12 μg·m^−3^ from 2008 to 2012. From 2012 to 2013, the concentration of SO_2_ and NO_2_ increased significantly, from, respectively, 12.73 ± 8.28 μg·m^−3^ to 22.68 ± 9.25 μg·m^−3^, and from 14.51 ± 6.92 μg·m^−3^ to 24.12 ± 7.97 μg·m^−3^, then decreased to 7.79 ± 4.96 μg·m^−3^ and 10.27 ± 4.88 μg·m^−3^ in 2015, and then stabilized to 2018. The average O_3_ concentration in Aba increased from 66.56 ± 14.26 μg·m^−3^ to 86.37 ± 23.17 μg·m^−3^ from 2015 to 2018. The annual average concentration of NO_2_ in Ganzi was always higher than that of SO_2_ from 2008 to 2018, but the annual variations of SO_2_ and NO_2_ were relatively close. SO_2_ wavelike variation went up from 2008 to 2013, then went down, then rose again from 2015 to 2016, then fell again; the lowest concentration was 9.05 ± 7.81 μg·m^−3^ in 2008, and the highest concentration was 22.62 ± 9.74 μg·m^−3^ in 2016. The lowest concentration of NO_2_ in Ganzi was 15.77 ± 8.72 μg·m^−3^ in 2008, and the highest concentration was 32.83 ± 8.96 μg·m^−3^ in 2012. Overall, SO_2_ and NO_2_ in Ganzi showed an upward and then a downward trend, and the annual average concentrations of 2008 and 2018 were relatively close. The average O_3_ concentration in Ganzi dropped from 62.47 ± 26.75 μg·m^−3^ in 2015 to 52.64 ± 19.97 μg·m^−3^ in 2016, and then rose to 95.45 ± 22.86 μg·m^−3^ in 2018.

The biggest difference between annual variation characteristics of SO_2_ and NO_2_ in the economic and ecological zones was that the annual average concentration of SO_2_ was almost always higher than that of NO_2_. In 2008–2016, the mean SO_2_ concentration in Liangshan was always higher than that of NO_2_. The mean SO_2_ concentration increased from 35.84 ± 16.76 μg·m^−3^ in 2008 to 43.41 ± 16.72 μg·m^−3^ in 2013 and then fell irregularly to 16.44 ± 4.71 μg·m^−3^ in 2018. In general, SO_2_ in Liangshan showed a downward trend, and the concentration in 2018 was 54.13% lower than that in 2008. NO_2_ wavelike variation in Liangshan increased from 21.82 ± 5.15 μg·m^−3^ in 2008 to 29.3 ± 5.95 μg·m^−3^ in 2012, (34.4%), and then fell to 20.52 ± 6.62 μg·m^−3^ in 2018. The annual average concentration of O_3_ in Liangshan showed a similar trend to Ganzi, first decreasing from 99.93 ± 27.66 μg·m^−3^ in 2015 to 92.49 ± 27.47 μg·m^−3^ in 2016, and then rising to 98.68 ± 2 8.85 μg·m^−3^ in 2018. Panzhihua has the highest urbanization among the four regions and the largest change range of SO_2_ annual average concentration. The SO_2_ concentration in Panzhihua was always higher than that of NO_2_ in 2008–2018. The average concentration of SO_2_ increased from 76.08 ± 32.77 μg·m^−3^ in 2008 to 86.52 ± 33.16 μg·m^−3^ in 2012, then fell sharply to 33.55 ± 14.65 μg·m^−3^ in 2015, then increased slightly to about 39 μg·m^−3^ in 2018. The average concentration of NO_2_ in Panzhihua decreased from 44.47 ± 12.01 μg·m^−3^ in 2008 to 31.82 ± 9.67 μg·m^−3^ in 2015, (28.45%), and then went up to 38.57 ± 10.49 μg·m^−3^ in 2018. The annual variation of O_3_ in Panzhihua showed a trend of first decreasing and then increasing, from 90.34 ± 22.89 μg·m^−3^ in 2014 to 75.79 ± 26.38 μg·m^−3^ in 2016, and then recovering to 93.05 ± 33.49 μg·m^−3^ in 2018.

In general, the annual variation of gas pollutants in the Western Sichuan Plateau, a relatively clean area, was different from that in large and medium-sized cities. The overall variations of SO_2_ and NO_2_ in the Western Sichuan Plateau were not very large, showing a trend of first increasing and then decreasing.

Overall, SO_2_ and NO_2_ in Aba, Ganzi, and Liangshan showed a small change range, and both showed a trend of first increasing and then decreasing in 2008–2018. However, after China’s 11th Five-Year Plan (2006–2010), SO_2_ concentration in large and medium-sized cities [14,29,30] showed an obvious downward trend, while NO_2_ showed a fluctuating or rising trend. The significant decrease of SO_2_ concentration in Panzhihua from 2012 to 2015 was also related to the emission reduction measures in China’s 12th Five-Year Plan (2011–2015). This shows that after the 11th Five-Year Plan, the emission reduction requirements for SO_2_ and NO_2_ proposed by the government had no obvious impact on the relatively clean Western Sichuan Plateau. The annual variations of O_3_ in the ecological and economic zones of the Western Sichuan Plateau were like those in Eastern China. Li et al. [20] showed that O_3_ increased significantly during the period of 2013–2017 in the North China Plain, the Pearl River Delta, Chengdu-Chongqing urban agglomeration, and Southeastern China, while the O_3_ pollution in parts of the northwest, southwest, and northeast of the country was relatively light.

**Figure 4:**
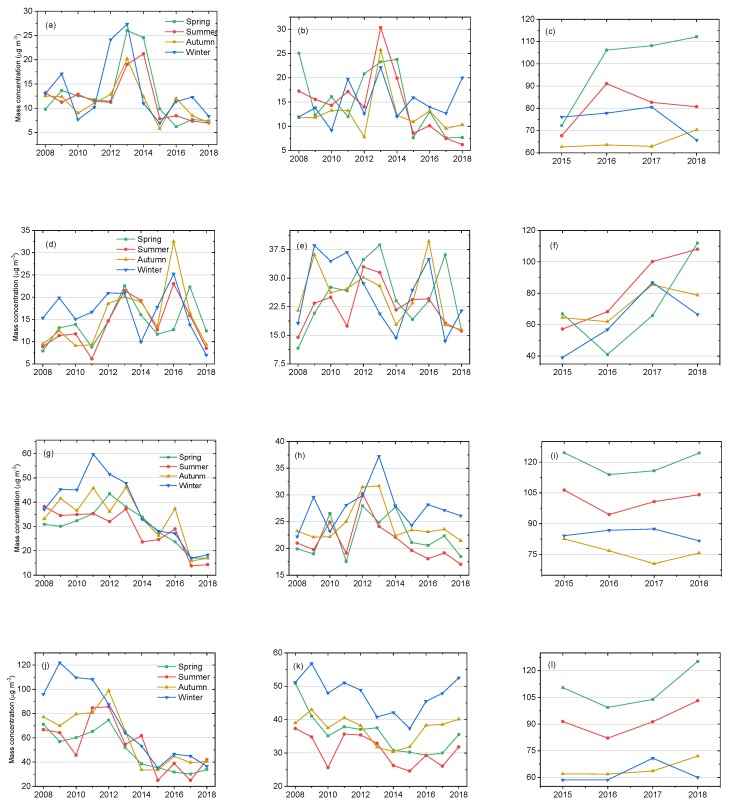
Annual variations of three air pollutants in different seasons: (**a**) SO_2_, (**b**) NO_2_, and (**c**) O_3_ in Aba; (**d**) SO_2_, (**e**) NO_2_, and (**f**) O_3_ in Ganzi; (**g**) SO_2_, (**h**) NO_2_, and (**i**) O_3_ in Liangshan; (**j**) SO_2_, (**k**) NO_2_, and (**l**) O_3_ in Panzhihua.

Figure 4 shows the annual variation characteristics of the average concentrations of SO_2_, NO_2_, and O_3_ in different seasons in the ecological and economic zones of Western Sichuan Plateau. Figure 4a–c show the annual variations of SO_2_, NO_2_, and O_3_ in different seasons in Aba. The annual changes of SO_2_ in spring and winter were more significant, rising first and then falling, reaching the maximum value in 2013. NO_2_ increased first and then decreased in all seasons, with the largest change in summer, reaching a maximum of 30.33 μg·m^−3^ in 2013. In both summer and winter, O_3_ showed an upward and then a downward trend, while in spring, it showed a very significant upward trend, with an increase of about 55.4% from 2015 to 2018. Figure 4d–f show the annual variations of seasonal average concentrations of SO_2_, NO_2_, and O_3_ in Ganzi. In the autumn, SO_2_ showed the most significant annual change, rising from 9.4 μg·m^−3^ in 2008 to 31.5 μg·m^−3^ in 2016, and finally falling to 9.3 μg·m^−3^ in 2018. NO_2_ fluctuated in all seasons, with the largest change in spring, increasing about 233.6% from 2008 to 2013, and decreasing about 58.1% from 2013 to 2018. In spring and autumn, O_3_ showed a downward and then an upward trend, while in summer, it showed a significant upward trend, with an increase of about 88.8% from 2015 to 2018. Both SO_2_ (Figure 4g) and NO_2_ (Figure 4h) in Liangshan showed the most obvious annual change range in winter; SO_2_ and NO_2_ reached the maximum value in 2011 and 2013, respectively. In spring, summer, and autumn, O_3_ in Liangshan (Figure 4i) showed a trend of first declining and then rising, while in winter it showed a trend of first rising and then falling, but the change range was small, decreasing by 5.8 μg·m^−3^ in 2017–2018. The seasonal average concentration of SO_2_ in Panzhihua (Figure 4j) showed the largest change in winter, with a decrease of 69.9% from 121.9 μg·m^−3^ in 2009 to 36.6 μg·m^−3^ in 2018. NO_2_ in Panzhihua (Figure 4k) showed a trend of first declining and then rising in all four seasons; the change ranges of spring and winter were large, and the seasonal average concentration in spring and winter reached the minimum value in 2016 and 2015, respectively. O_3_ in Panzhihua (Figure 4l) showed a trend of first declining and then rising in all four seasons, with a large change range in spring and summer, and an increase of about 26% in spring from 2016 to 2018.

**Figure 5:**
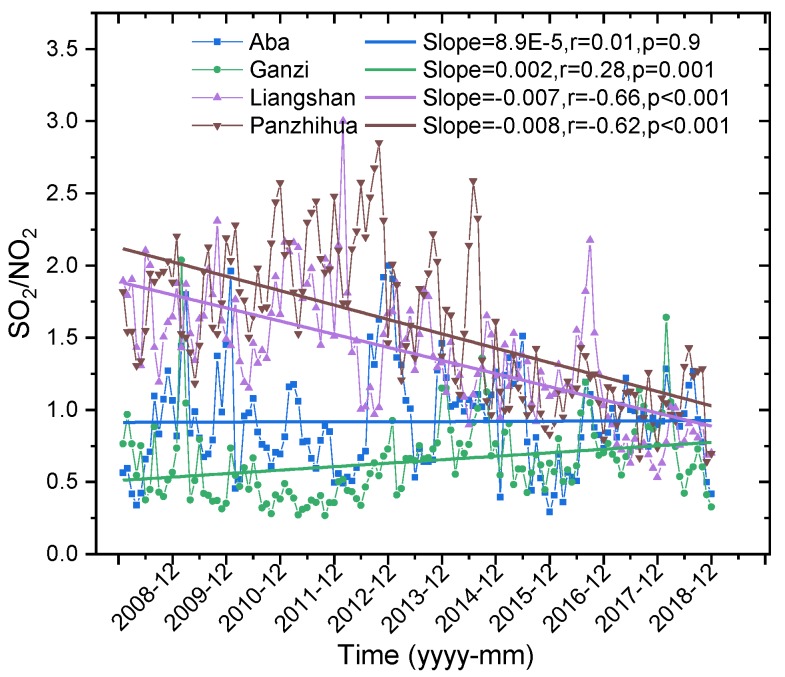
Variations of concentration ratio of SO_2_ to NO_2_ in Aba, Ganzi, Liangshan, and Panzhihua from 2008 to 2018.

The average mass concentration ratio of SO_2_ and NO_2_ (SO_2_/NO_2_) can represent the energy structure characteristics of a local region [31]. For example, in regions with more developed industries, a large amount of sulfur-containing fuel is consumed and SO_2_ is emitted, thus, SO_2_/NO_2_ is larger. If an area has a mass of cars and emits more NO_x_, the ratio is relatively small. In the 1990s, SO_2_/NO_2_ in North America and some eastern and central European countries exceeded 1, indicating that these regions had developed industries and the emissions were mainly SO_2_ [31]. Figure 5 shows the change characteristics of SO_2_/NO_2_ based on monthly average concentration in the ecological and economic zones of Western Sichuan Plateau from 2008 to 2018. As can be seen, SO_2_/NO_2_ of Aba and Ganzi in the ecological zone showed an upward trend, while SO_2_/NO_2_ of Liangshan and Panzhihua in the economic zone showed a downward trend. From 2008 to 2018, SO_2_/NO_2_ of Aba fluctuated and showed a slight upward trend, with an average increase of 8.9 × 10^−5^/month. Overall, SO_2_/NO_2_ of Ganzi was smaller than that of Aba, and the overall SO_2_/NO_2_ fluctuated and had a more obvious upward trend, with an average increase of 0.002/month, and SO_2_/NO_2_ stayed below 1.0 most of the time. SO_2_/NO_2_ in both Liangshan and Panzhihua showed an obvious downward trend, with an average decline of −0.007/month in Liangshan and a slightly higher rate in Panzhihua, −0.008/month. According to the annual average concentration ratio of SO_2_ and NO_2_ (not shown), before 2017, the annual SO_2_/NO_2_ in Liangshan was greater than 1.0, and the maximum value (about 1.88) appeared in 2011, while in 2017 and 2018, SO_2_/NO_2_ was less than 1.0 (0.73 and 0.80, respectively). The annual SO_2_/NO_2_ in Panzhihua was higher than that in Liangshan; from 2008 to 2012, it showed a quasi-linear growth trend, increasing from 1.71 in 2008 to 2.17 in 2012, and then significantly decreasing, reaching the lowest value of about 0.99 in 2017.

The rising trend of SO_2_/NO_2_ in the Western Sichuan Plateau ecological area and the declining trend in the economic area indicates that the energy consumption structure of these two areas is quite different. The rising trend of the ecological area indicates that industrial SO_2_ emission of in the area have increased in the past 11 years compared with NO_x_ emissions from transportation. However, the declining trend of SO_2_/NO_2_ in the economic zone in the past decade is similar to that of some large and medium-sized cities in eastern China, indicating that the SO_2_ emission reduction effect in this region was relatively obvious, while the NO_x_ emission reduction effect was not significant. It is worth noting that China’s 11th Five-Year Plan (2011–2015) put forward specific SO_2_ and NO_x_ emission reduction targets, since SO_2_/NO_2_ in most Chinese cities showed a declining trend, and SO_2_/NO_2_ in the ecological zone of Western Sichuan Plateau, which is cleaner than the large and medium-sized cities, showed an increasing trend. This suggests that the SO_2_ pollution level in clean areas is relatively light, but its growth trend cannot be ignored.

**Figure 6:**
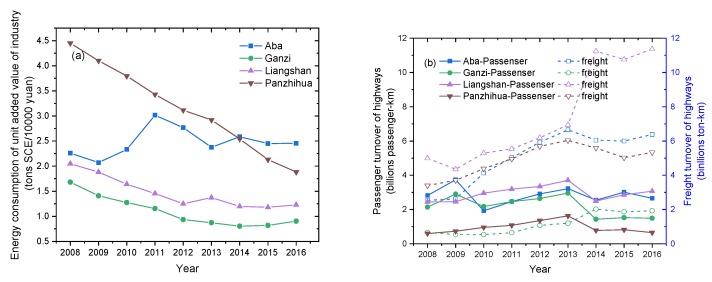
(**a**) Energy consumption per unit industrial added value and (**b**) highway passenger and freight turnover in Aba, Ganzi, Liangshan, and Panzhihua in 2008–2016.

Due to the lack of emissions data of SO_2_ and NO_x_ in the ecological and economic zones of Western Sichuan Plateau, the energy consumption of unit added industrial value and the passenger and freight turnover of highways were selected to indirectly characterize the emission change characteristics of SO_2_ and NO_x_, respectively. As can be seen from Figure 6a, from 2008 to 2016, the energy consumption of unit added industrial value in Aba first increased and then decreased, while other regions showed a significant downward trend, indicating that industrial consumption of standard coal equivalent (SCE) in other regions except Aba declined year by year. Compared with the change characteristics of SO_2_ concentration from 2008 to 2018 (Figure 3), it was found that the variations of SO_2_ are not consistent with industrial emissions, indicating that in these relatively clean areas, other sources besides industrial emissions will also contribute significantly to SO_2_; for instance, biomass combustion in Qinghai Plateau [32] makes an important contribution to SO_2_. The passenger and freight turnover of highways (Figure 6b) shows an overall upward trend, first increasing and then decreasing before and after 2013, which is close to the changing trend of NO_2_ concentration (Figure 3), indicating that motor vehicle emissions made a prominent contribution to the change in NO_2_ concentration.

### 3.2. Effects of Meteorology on SO_2_, NO_2_ and O_3_

**Figure 7:**
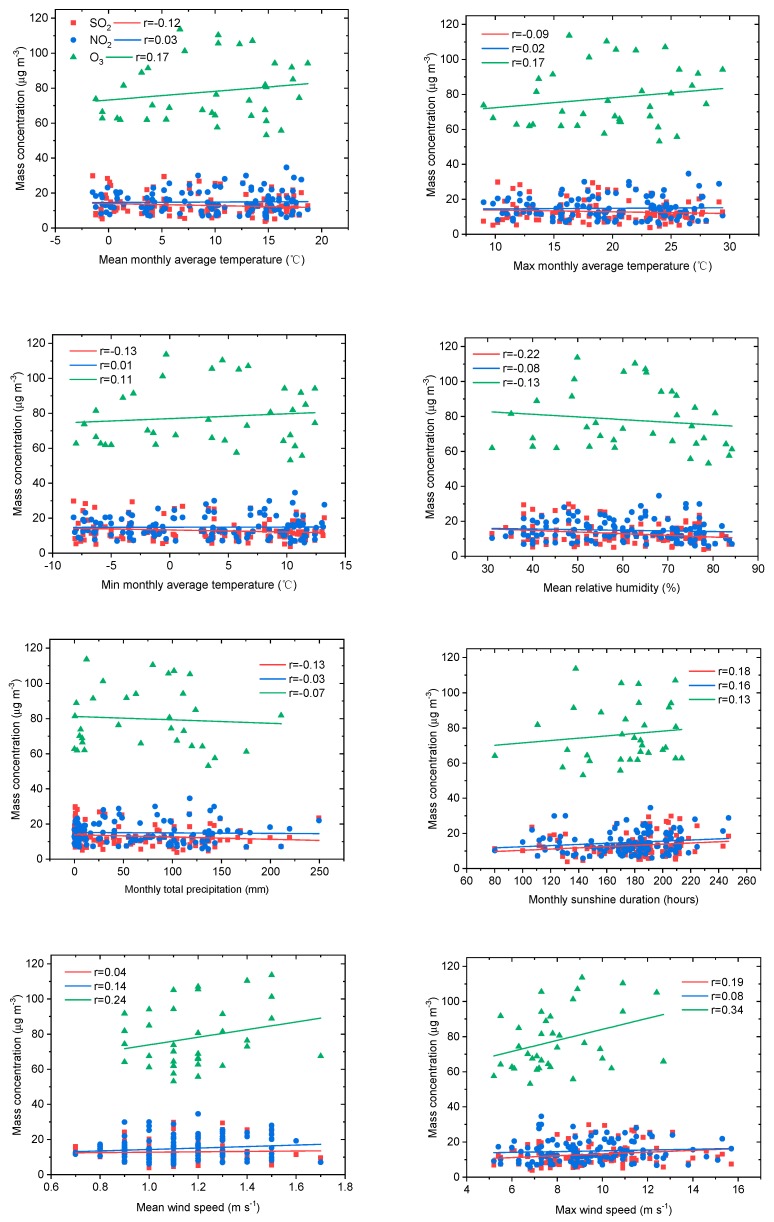
Relationships between SO_2_, NO_2_, O_3_ and routine meteorological parameters (mean monthly average temperature, maximum and minimum monthly average temperature, mean relative humidity, monthly total precipitation, monthly sunshine duration, mean wind speed, and maximum wind speed) in Aba of the ecological zone of Western Sichuan Plateau.

Emissions and meteorological conditions are predominant in the air pollutant trend; emissions exceeding atmospheric environmental capacity is the basic cause of air pollution [33,34]. In order to analyze the influence of meteorological factors on gaseous air pollutants in the ecological and economic zones of Western Sichuan Plateau, the relationships between SO_2_, NO_2_, O_3_, and routine meteorological parameters (mean monthly average temperature, maximum and minimum monthly average temperature, mean relative humidity, monthly total precipitation, monthly sunshine duration, mean wind speed, and maximum wind speed) were discussed by using monthly mean meteorological data from 2008 to 2017. Figure 7 shows these relationships in Aba, in the ecological zone. SO_2_ and NO_2_ concentrations were less dependent on meteorological parameters in the ecological zone with less anthropogenic emission. The relationship between O_3_ and meteorological parameters in Figure 7 implies that a high concentration of O_3_ was more likely to form under the meteorological conditions of high temperature, low relative humidity, long sunshine duration, and especially high wind speed.

**Figure 8:**
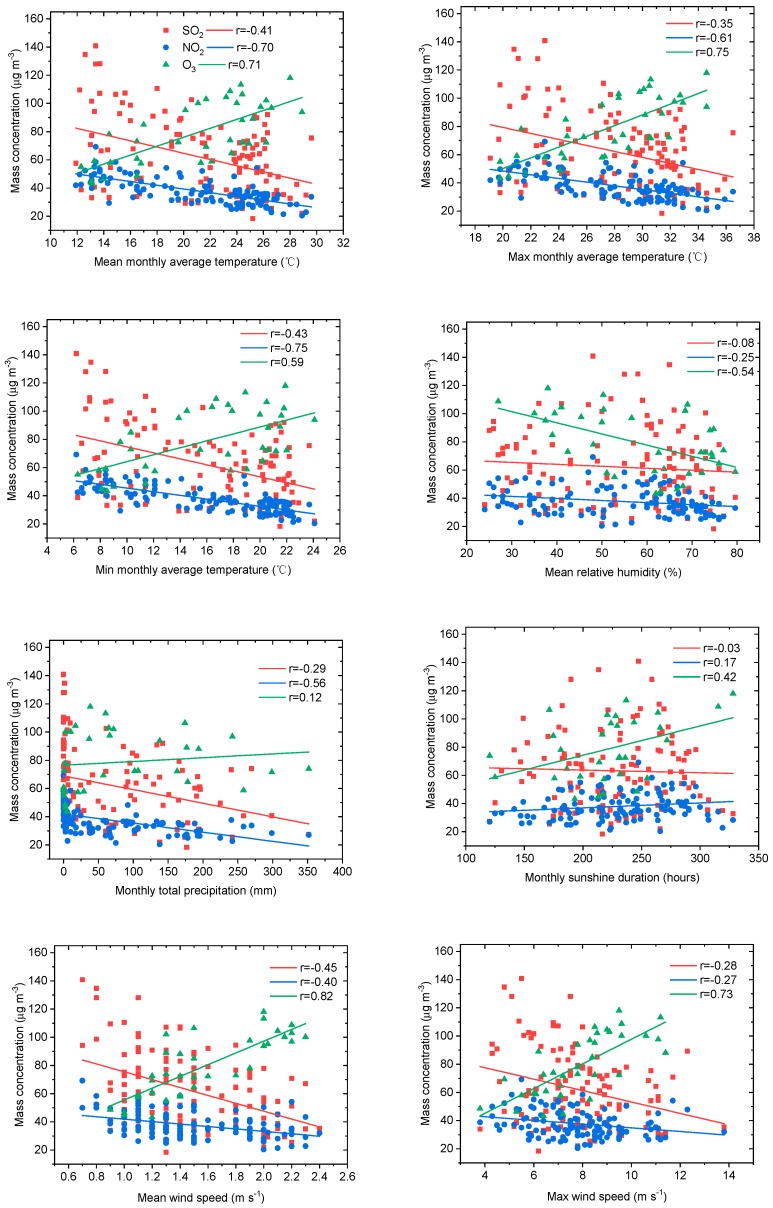
Relationships between SO_2_, NO_2_, O_3_, and routine meteorological parameters (mean monthly average temperature, maximum and minimum monthly average temperature, mean relative humidity, monthly total precipitation, monthly sunshine duration, mean wind speed, and maximum wind speed) in Panzhihua of the economic zone of Western Sichuan Plateau.

The relationships of SO_2_, NO_2_, O_3_, and meteorological parameters in Panzhihua of the economic zone of Western Sichuan Plateau are shown in Figure 8. In the economic zone, with more man–made emissions than the ecological zone, gaseous pollutants are more significantly affected by meteorological parameters with a higher coefficient of determination. SO_2_ and NO_2_ decreased with increased temperature, humidity, precipitation, and wind speed. O_3_ was positively correlated with temperature, sunshine duration, and wind speed and negatively correlated with relative humidity. The dependence of gaseous pollutants on meteorological factors was more prominent in the economic zone than in the ecological zone, which may have to do with local emissions and special topography [21].

### 3.3. Characteristics of SO_2_, NO_2_, and O_3_

**Figure 9:**
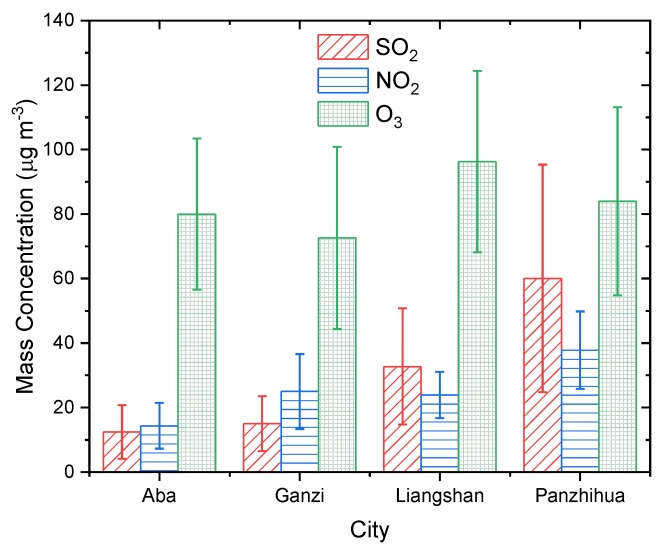
Multi-year mean mass concentrations of SO_2_, NO_2_, and O_3_ in Aba, Ganzi, Liangshan, and Panzhihua.

Figure 9 shows the multi-year average mass concentrations of SO_2_, NO_2_, and O_3_ in the Western Sichuan Plateau ecological and economic zones. The average mass concentrations of O_3_ in Aba, Ganzi, Liangshan, and Panzhihua were significantly higher than that of SO_2_ and NO_2_. Liangshan suffered the most serious O_3_ pollution of the four regions, with a mean mass concentration of 96.25 ± 28.17 μg·m^−3^, followed by Panzhihua, 83.96 ± 29.17 μg·m^−3^. O_3_ pollution in the ecological region, at higher altitude, was lighter than in the economic zone, with mean mass concentrations of 79.99 ± 23.41 μg·m^−3^ and 72.61 ± 28.28 μg·m^−3^, respectively, in Aba and Ganzi. The mean mass concentrations of SO_2_ and NO_2_ in the ecological zone were lower than in the economic zone. The mean mass concentration of SO_2_ in Aba and Ganzi was similar, at 12.39 ± 8.31 μg·m^−3^ and 15.02 ± 8.51 μg·m^−3^, respectively, and in Panzhihua, it was significantly higher than in Liangshan, at 60.05 ± 35.25 μg·m^−3^ and 32.73 ± 18.02 μg·m^−3^, respectively. Among the four regions, Panzhihua has the most serious SO_2_ pollution, and Aba has the least, the multi-year average concentration of SO_2_ in Panzhihua is about five times that in Aba. For NO_2_, the average mass concentration was highest in Panzhihua, at 37.81 ± 11.98 μg·m^−3^ and lowest in Aba, at 14.37 ± 7.07 μg·m^−3^. The level of NO_2_ pollution in Ganzi and Liangshan was moderate, and the average concentration of NO_2_ was similar, 24.99±11.59 μg·m^−3^ and 23.9 ± 7.17 μg·m^−3^, respectively. Zhao et al. [21] analyzed the characteristics of six criteria for air pollutants in the Sichuan basin in 2015–2017, and showed that the average SO_2_ concentration in Chengdu, the provincial city, was close to that in the ecological zone due to effective desulfurization measures and strict SO_2_ emission reduction requirements, while the average NO_2_ and O_3_ concentration in Chengdu was significantly higher than that in the ecological and economic zones in this study.

**Figure 10:**
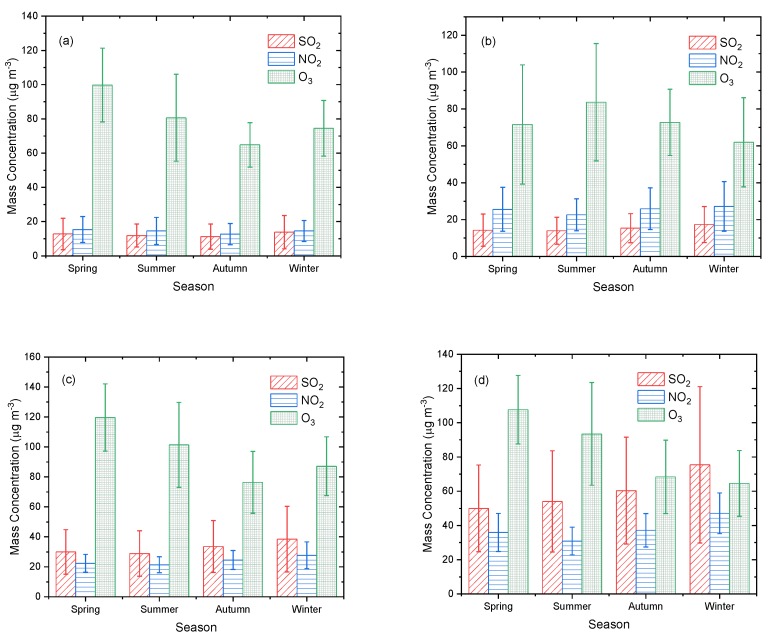
Seasonal mean mass concentrations of SO_2_, NO_2_, and O_3_ in (**a**) Aba, (**b**) Ganzi, (**c**) Liangshan, and (**d**) Panzhihua.

Figure 10 shows the seasonal average mass concentrations of SO_2_, NO_2_, and O_3_ in the ecological and economic zones of Western Sichuan Plateau. The seasonal variation of O_3_ is more notable than that of SO_2_ or NO_2_. The seasonal distribution of O_3_ in Aba and Liangshan is similar, with the highest concentration in spring and the lowest in autumn, presenting a seasonal distribution as spring > summer > winter > autumn. The average concentration of O_3_ in Aba was 99.8 ± 21.54 μg·m^−3^ and 64.83 ± 12.95 μg·m^−3^, and that in Liangshan was 119.64 ± 22.47 μg·m^−3^ and 76.42 ± 20.63 μg·m^−3^ in spring and autumn, respectively. The seasonal distribution of O_3_ in Ganzi was summer > autumn > spring > winter, with an average concentration of 83.67 ± 31.85 μg·m^−3^ in summer and 64.94 ± 24.14 μg·m^−3^ in winter. The concentration of O_3_ in Panzhihua reached its highest in spring 107.64 ± 19.97 μg·m^−3^, and its lowest in winter, 64.61 ± 19.2 μg·m^−3^, indicating a seasonal distribution of spring > summer > autumn > winter.

The average concentration of SO_2_ in Ganzi and Liangshan was the same, with a seasonal distribution of winter > autumn > spring > summer. The average concentration was 17.4 ± 9.7 μg·m^−3^ and 13.94 ± 7.28 μg·m^−3^ in Ganzi and was 38.46 ± 21.88 μg·m^−3^ and 28.87 ± 15.15 μg·m^−3^ in Liangshan in winter and summer, respectively. The maximum concentration of SO_2_ in Aba occurred in winter, while the minimum concentration occurred in autumn, at 13.84 ± 9.74 μg·m^−3^ and 11.24 ± 7.38 μg·m^−3^; the seasonal distribution was winter > spring > summer > autumn. The mean concentration of SO_2_ in Panzhihua was the highest in winter, 75.44 ± 45.67 μg·m^−3^, and the lowest in spring 49.99 ± 25.32 μg·m^−3^.

Ganzi, Liangshan, and Panzhihua had the same NO_2_ seasonal distribution of winter > autumn > spring > summer. The difference in NO_2_ mean concentration in Ganzi and Liangshan between the four seasons was not significant; the maximum and minimum concentrations in Ganzi were 27.16 ± 13.5 μg·m^−3^ and 22.66 ± 8.68 μg·m^−3^, respectively, and the difference between winter and summer was about 4.5 μg·m^−3^. The maximum and minimum NO_2_ mean concentrations in Liangshan were 27.72 ± 8.93 μg·m^−3^ and 21.37 ± 5.31 μg·m^−3^, respectively, and the difference in concentration between winter and summer was about 6.3 μg·m^−3^. The average concentration of NO_2_ in Panzhihua in the four seasons was significantly different; the maximum concentration was 47.14 ± 11.88 μg·m^−3^ in winter, the minimum concentration was 30.89 ± 8.13 μg·m^−3^ in summer, and the difference was about 16.2 μg·m^−3^. The seasonal distribution of NO_2_ in Aba was different from the other regions, with an average concentration of 15.39 ± 7.57 μg·m^−3^ in spring and the lowest concentration of 12.71 ± 6.19 μg·m^−3^ in autumn.

**Figure 11:**
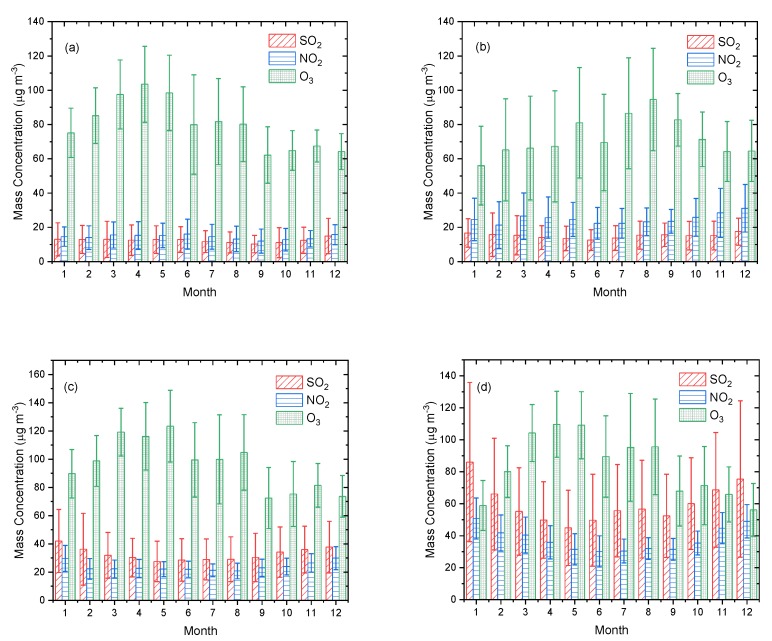
Monthly mean mass concentrations of SO_2_, NO_2_, and O_3_ in (**a**) Aba, (**b**) Ganzi, (**c**) Liangshan, and (**d**) Panzhihua.

Figure 11 shows the monthly distribution characteristics of the three pollutants. High concentrations of O_3_ in Ganzi appeared in July, August, and September, and in Aba, Liangshan, and Panzhihua in March, April, and May. The maximum O_3_ concentration in Ganzi was 94.63 ± 29.89 μg·m^−3^ in August, and the minimum was 56.05 ± 22.94 μg·m^−3^ in January. The maximum and minimum concentrations of O_3_ in Aba occurred in April and September, respectively, at 103.46 ± 22.15 μg·m^−3^ and 62.16 ± 16.47 μg·m^−3^. The maximum concentration of O_3_ in Liangshan occurred in May at 123.40 ± 25.3 μg·m^−3^ and the minimum concentration of 72.49 ± 21.66 μg·m^−3^ in September. The maximum and minimum concentrations of O_3_ in Panzhihua occurred in April and December, at 109.65 ± 20.66 μg·m^−3^ and 56.17 ± 16.49 μg·m^−3^, respectively. The differences between maximum and minimum monthly concentrations in the economic zone were significantly higher than in the ecological zone, among which the difference in Panzhihua was the largest, about 53.48 μg·m^−3^, and the difference in Ganzi was the smallest, about 38.5 μg·m^−3^, indicating that the monthly change of O_3_ with a longer residence time was more significant in the area with more artificial emissions than in the clean area with less artificial emissions.

The monthly mean concentrations of SO_2_ in the ecological zone did not change significantly. The maximum and minimum concentrations in Aba appeared in December and September, respectively, and the difference was about 4.76 μg·m^−3^, while the maximum and minimum concentrations in Ganzi appeared in December and June, respectively, and the difference was about 4.9 μg·m^−3^. Artificial emissions were more prominent in the economic zone, and the monthly variations of average concentrations of SO_2_ were more obvious. The maximum and minimum SO_2_ concentration in Liangshan occurred in January and May, with a difference of 14.43 μg·m^−3^, and in Panzhihua occurred in January and May, respectively, at 86.01 ± 49.78 μg·m^−3^ and 44.96 ± 23.54 μg·m^−3^, with a difference of 41.05 μg·m^−3^. The range of variation of monthly average NO_2_ concentration in Aba was the lowest in the ecological and economic zones; the maximum and minimum concentrations appeared in June and September, with a difference of 6.05 μg·m^−3^, and the maximum and minimum concentrations in Ganzi appeared in December and February, with a difference of 9.82 μg·m^−3^, respectively. The monthly average NO_2_ concentration in Panzhihua showed the most prominent variation characteristics, with a mean maximum of 50.82 ± 12.79 μg·m^−3^ in January, minimum of 30.21 ± 9.79 μg·m^−3^ in June, and a difference of 20.61 μg·m^−3^. The maximum and minimum monthly average concentrations of NO_2_ in Liangshan occurred in December and August, respectively, with a difference of 9.01 μg·m^−3^.

### 3.4. Correlation between SO_2_, NO_2_, and O_3_

**Figure 12:**
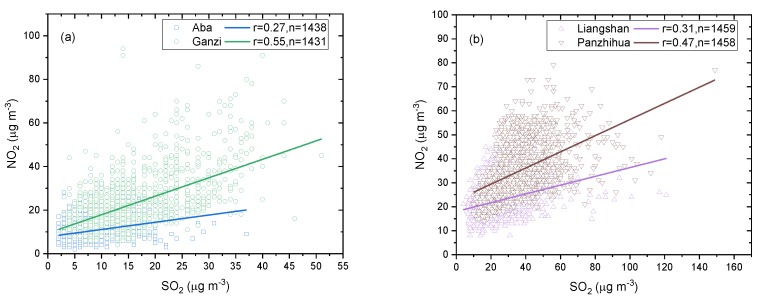
Correlation between SO_2_ and NO_2_ in the (**a**) ecological zone and (**b**) economic zone in 2015–2018.

It is generally believed that CO and NO_x_ pollutants are discharged by mobile sources, while SO_2_ and NO_x_ pollutants are discharged by point sources [35]. Since mobile sources do not discharge a large amount of SO_2_ and point sources produce both SO_2_ and NO_2_, if the correlation between SO_2_ and NO_2_ is high, the point source is more prominent. On the other hand, if the correlation is low, the mobile source is more prominent. Figure 12 shows the correlation between SO_2_ and NO_2_ in Aba, Ganzi, Liangshan, and Panzhihua. SO_2_ and NO_2_ in the ecological and economic zones show a positive correlation with a significance test of 99% confidence. The correlation between SO_2_ and NO_2_ in Ganzi is the highest, with a Pearson coefficient r 0.55, followed by Panzhihua, with r = 0.47, which indicates that the emission characteristics of SO_2_ and NO_2_ in these two regions are relatively similar, SO_2_ and NO_2_ are discharged mainly from point sources and point sources contribute more to local air pollutants than mobile sources. The lowest correlation between SO_2_ and NO_2_ is found in Aba, with a correlation coefficient of 0.27, while that in Liangshan is slightly higher, with r = 0.31, indicating that the contribution of point sources to SO_2_ and NO_2_ pollution in these two areas is not very prominent. In general, although SO_2_ and NO_2_ in the ecological and economic zones were responsible less pollution than in other large and medium-sized cities in China, the emission source characteristics of SO_2_ and NO_2_ in this region were different from those of the other cities. Mao et al. [36] reported a strong positive correlation between SO_2_ and NO_2_ in Chongqing, Wuhan, and Nanjing, three metropolises along the Yangtze River, indicating the similar origins and elimination processes of SO_2_ and NO_2_.

**Figure 13:**
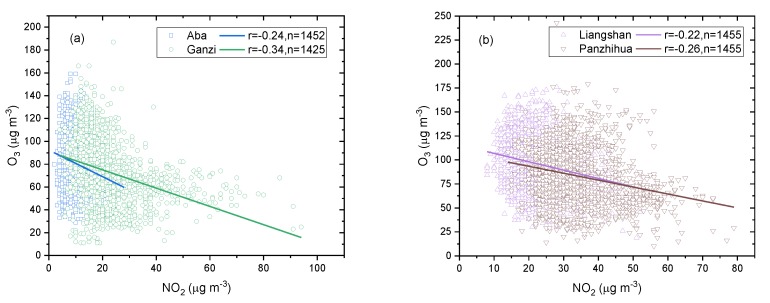
Correlation between NO_2_ and O_3_ in the (**a**) ecological zone and (**b**) economic zone in 2015–2018.

O_3_ is an important secondary gaseous pollutant in the urban atmosphere, and NO_x_ plays a very important role in its formation as the main precursor. The formation of surface O_3_ is highly dependent on solar radiation intensity, the absolute concentration of O_3_ and volatile organic compounds (VOCs), and the ratio of O_3_ to VOCs, while the local surface concentration of O_3_ is affected by meteorological elements, local precursor emissions, and the close and long-distance transport of O_3_ and precursor [37,38,39]. As shown in Figure 13a,b, the correlations between NO_2_ and O_3_ in the ecological and economic zones of Western Sichuan Plateau were analyzed by using the daily mass concentration from 2015 to 2018. The correlations between NO_2_ and O_3_ in the four regions were negatively correlated, and all of them passed the significance test of 99% confidence. Among the four regions, Ganzi has the highest Pearson correlation coefficient (−0.34), indicating that this region is where NO_2_ contributes the most to O_3_ generation as a precursor. Aba, Liangshan, and Panzhihua showed a relatively low correlation between NO_2_ and O_3_, with a Pearson correlation coefficient of −0.24, −0.22, and −0.26, respectively. The correlations between NO_2_ and O_3_ in the metropolitan areas of China, such as the Beijing–Tianjin–Hebei region, Yangtze River Delta, Pearl River Delta, and Chengdu–Chongqing city cluster [2,36,40] are significantly higher than in the ecological and economic zones of the Western Sichuan Plateau in our study, indicating that NO_2_ contributes more to O_3_ in large and medium-sized urban areas with high NO_2_ emissions. The contribution of local precursors of O_3_ can better explain the formation of O_3_, while in regions with less NO_2_ emission, O_3_ may be more affected by the regional background. de Souza et al. [41] also indicated that when the correlation between NO_2_ and O_3_ is high, the local precursor plays a leading contributory role, and when the correlation is low, the variation of O_3_ concentration is mainly affected by the O_3_ concentration in the regional background.

## 4. Conclusions

Based on daily average mass concentration data of SO_2_ and NO_2_ from 2008 to 2018 and O_3_ from 2015 to 2018 in Aba, Ganzi, Liangshan, and Panzhihua in Western Sichuan Plateau, the characteristics and change trend of the three gas pollutants and their correlations in the relatively clean ecological and economic zones were analyzed. The main conclusions are as follows:

(1) On the whole, the change ranges of SO_2_ and NO_2_ in Aba, Ganzi, and Liangshan were not large, and they all showed a trend of first increasing and then decreasing. The change range of SO_2_ in Panzhihua was the most obvious, and the decrease range was very significant from 2012 to 2015. During the period 2015–2018, except for Aba, O_3_ showed a trend of first declining and then rising in the other regions. SO_2_/NO_2_ in the economic zone of Western Sichuan Plateau showed a decreasing trend, while in the relatively clean ecological zone this ratio showed an increasing trend. Although the SO_2_ pollution level in the clean areas was relatively low, the growth trend could not be ignored. The dependence of SO_2_ and NO_2_ on routine meteorological parameters in the ecological zone was low. The influence of meteorological parameters on gaseous pollutants was more significant in the economic zone with high anthropometric emissions.
(2) O_3_ pollution in the Western Sichuan Plateau with less artificial emission was prominent, with the highest annual mean concentration in Liangshan, 96.25 ± 28.17 μg·m^−3^, and the lowest annual mean concentration in Ganzi, 72.61 ± 28.28 μg·m^−3^, indicating that O_3_ formation is promoted by strong solar radiation induced by smaller particle concentrations and more cloud-free days [42,43]. The pollution levels of SO_2_ and NO_2_ in the ecological zone were lower than those in the economic zone. The annual average concentration of SO_2_ in Panzhihua and Aba was the highest and lowest, at 12.39 ± 8.31 μg·m^−3^ and 60.05 ± 35.25 μg·m^−3^, respectively. Panzhihua had the highest annual average NO_2_ concentration, 37.81 ± 11.98 μg·m^−3^, while Aba had the lowest annual average concentration, 14.37 ± 7.07 μg·m^−3^. The seasonal and monthly variations of O_3_ in the four regions were more obvious than those of SO_2_ and NO_2_. The seasonal and monthly variations in the economic zone were higher than those in the ecological zone, indicating that man-made emissions have an important impact on the temporal distribution characteristics of air pollutants.
(3) The correlation between SO_2_ and NO_2_ in Ganzi was the highest, followed by Panzhihua; the emission characteristics of SO_2_ and NO_2_ in these two regions were similar, and point sources contributed more to local air pollutants than mobile sources. The correlation between SO_2_ and NO_2_ was the lowest in Aba, indicating that the contribution of point sources to SO_2_ and NO_2_ pollution was not prominent. Ganzi showed the highest correlation coefficient, suggesting that NO_2_ contribute the most to O_3_ in this region in the Western Sichuan Plateau, followed by Panzhihua, Aba, and Liangshan. The correlation between NO_2_ and O_3_ is significantly higher in large and medium-sized cities in China than in the ecological and economic zones of Western Sichuan Plateau, which indicates that local precursors contribute more to the formation of O_3_ in areas with high NO_2_ emissions, while in areas with low NO_2_ emissions, O_3_ is more significantly affected by the regional background.

## Figures and Tables

**Table 1:** Resident population, urbanization rate, population density, gross domestic product (GDP), possession of civil motor vehicles and area in the ecological and economic zones of Western Sichuan Plateau.

	Region Category	Administrative Area (km^2^)	Resident Population (10,000)	Urbanization Rate (%)	GDP (Billion Yuan)	Possession of Civil Motor Vehicles (10,000)
Aba	Ecological zone	83,016	93.46	37.86	28.13	10.5
Ganzi	Ecological zone	149,599	118.05	29.26	22.98	8.3
Liangshan	Economic zone	60,294	482.22	33.04	140.39	24.9
Panzhihua	Economic zone	7401	123.56	65.34	101.47	15.1
